# Functional classification of rice flanking sequence tagged genes using MapMan terms and global understanding on metabolic and regulatory pathways affected by *dxr* mutant having defects in light response

**DOI:** 10.1186/s12284-016-0089-2

**Published:** 2016-04-14

**Authors:** Anil Kumar Nalini Chandran, Gang-Seob Lee, Yo-Han Yoo, Ung-Han Yoon, Byung-Ohg Ahn, Doh-Won Yun, Jin-Hyun Kim, Hong-Kyu Choi, GynHeung An, Tae-Ho Kim, Ki-Hong Jung

**Affiliations:** Graduate School of Biotechnology & Crop Biotech Institute, Kyung Hee University, Yongin, 446-701 Republic of Korea; Molecular Breeding Division, National Academy of Agricultural Science, RDA, Jeonju, 560-500 Republic of Koreas; Genomics Division, National Academy of Agricultural Science, RDA, Jeonju, 560-500 Republic of Korea; R&D Coordination Division, Research Policy Bureau, RDA, Jeonju, 560-500 Republic of Korea; Planning & Coordination Division, National Academy of Agricultural Science, RDA, Jeonju, 560-500 Republic of Korea; Department of Medical Bioscience, Dong-A University, Busan, Republic of Korea

**Keywords:** *DXR*, Gene-indexed mutant, Functional genomics, MapMan analysis, Rice

## Abstract

**Background:**

Rice is one of the most important food crops for humans. To improve the agronomical traits of rice, the functions of more than 1,000 rice genes have been recently characterized and summarized. The completed, map-based sequence of the rice genome has significantly accelerated the functional characterization of rice genes, but progress remains limited in assigning functions to all predicted non-transposable element (non-TE) genes, estimated to number 37,000–41,000.

**Results:**

The International Rice Functional Genomics Consortium (IRFGC) has generated a huge number of gene-indexed mutants by using mutagens such as T-DNA, Tos17 and Ds/dSpm. These mutants have been identified by 246,566 flanking sequence tags (FSTs) and cover 65 % (25,275 of 38,869) of the non-TE genes in rice, while the mutation ratio of TE genes is 25.7 %. In addition, almost 80 % of highly expressed non-TE genes have insertion mutations, indicating that highly expressed genes in rice chromosomes are more likely to have mutations by mutagens such as T-DNA, *Ds*, *dSpm* and *Tos17*. The functions of around 2.5 % of rice genes have been characterized, and studies have mainly focused on transcriptional and post-transcriptional regulation. Slow progress in characterizing the function of rice genes is mainly due to a lack of clues to guide functional studies or functional redundancy. These limitations can be partially solved by a well-categorized functional classification of FST genes. To create this classification, we used the diverse overviews installed in the MapMan toolkit. Gene Ontology (GO) assignment to FST genes supplemented the limitation of MapMan overviews.

**Conclusion:**

The functions of 863 of 1,022 known genes can be evaluated by current FST lines, indicating that FST genes are useful resources for functional genomic studies. We assigned 16,169 out of 29,624 FST genes to 34 MapMan classes, including major three categories such as DNA, RNA and protein. To demonstrate the MapMan application on FST genes, transcriptome analysis was done from a rice mutant of 1-deoxy-D-xylulose 5-phosphate reductoisomerase (*DXR)* gene with FST. Mapping of 756 down-regulated genes in *dxr* mutants and their annotation in terms of various MapMan overviews revealed candidate genes downstream of *DXR*-mediating light signaling pathway in diverse functional classes such as the methyl-D-erythritol 4-phosphatepathway (MEP) pathway overview, photosynthesis, secondary metabolism and regulatory overview. This report provides a useful guide for systematic phenomics and further applications to enhance the key agronomic traits of rice.

**Electronic supplementary material:**

The online version of this article (doi:10.1186/s12284-016-0089-2) contains supplementary material, which is available to authorized users.

## Background

Rice (Oryza sativa) is one of the most important crops worldwide, as both a staple food and a model system for genomic research. The complete genome sequence is a basic resource to elucidate individual gene functions and initiate comparative genomics among crop species. As a monocot model, high-throughput omics data are available for rice at multiple levels including microarray and RNA-seq, proteomic data, protein-protein interactions, reactome, and genome-wide gene-indexed mutant collections (Chandran and Jung 2014). These data are helpful for precisely estimating detailed gene functions. The functions of more than 1,000 rice genes have been elucidated by genetic analysis with natural and gene-indexed mutants or transgenic approaches (Yamamoto et al. 2012). These genes constitute about 2.5 % of the predicted loci in the Rice Annotation Project Database (RAP-DB) (Tanaka et al. 2008), indicating that the functions of most rice genes have yet to be elucidated. Progress in rice functional genomics requires high-throughput omics data from multiple levels. The gene-indexed mutant population is a key resource for functional genomics and the International Rice Functional Genomics Consortium (IRFGC) has generated gene-indexed mutants that are available to study the functions of around 30,000 genes.

Two main obstacles are expected to limit the progress of current functional genomic analyses with gene-indexed mutants. One obstacle is gene redundancy arising from large segmental gene duplications and tandem duplications (Yu et al. 2005). The redundancy is partial or uneven, depending on the similarities in expression of duplicated genes (Briggs et al. 2006). The other obstacle is the limited information available to study gene function. MapMan is a useful tool to provide global views of diverse aspects of high-throughput data and can be used to functionally classify rice genes with flanking sequence tag (FST) mutants for systematic functional studies (Thimm et al. 2004; Usadel et al. 2009; Jung and An 2012).

In this report, we tried to systematically assign functions to all predicted rice genes with FSTs. For this purpose, a total of 246,566 FSTs from various mutant resources worldwide along with new FSTs we identified with Ds insertions were collected and mapped to the rice genome (Chandran and Jung 2014). We analyzed the expression patterns of FST genes using Agrobacterium infected callus samples at different time points. Next, functional assignment was done for 29,624 rice FST genes based on functional classification information from the MapMan Toolkit. This information can be used for systematic functional studies on selected metabolic pathways or functional modules. As a case study of metabolic pathways or functional modules assessment using MapMan tool, we carried out transcriptome profiling of rice mutant leaves in 1-deoxy-D-xylulose 5-phosphate reductoisomerase (*DXR)* gene compared to its wild type control. Estimation and further annotating of these down regulated genes in *dxr* mutant using MapMan classification informs diverse routes for the regulation or metabolism of the light response pathway and associated genes. Use of additional FSTs will clarify the functionality of the routes associated with *DXR*. Gene Ontology (GO) assignment on FSTs partially supplements the limitation of MapMan annotation.

## Results and discussion

### Current state of rice gene-indexed mutants by transposons and T-DNA insertions

**Fig 1:**
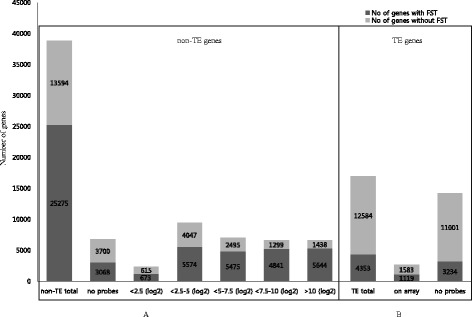
Distribution of T-DNA insertions according to expression levels in callus samples after agro-inoculation. **a** FST information and probe level expression distribution of non-TE genes. **b** FST information of TE genes

To assign functions to rice genes for the post-genomic era, we collected gene-indexed rice mutants available worldwide: POSTECH-RISD (Jeon et al. 2000), UCD (Kumar et al. 2005), GSNU (Kim et al. 2004), CIRAD (http://oryzatagline.cirad.fr/), TRIM (http://trim.sinica.edu.tw/), NIAS (https://tos.nias.affrc.go.jp/), RMD (http://rmd.ncpgr.cn/), SHIP T-DNA (http://signal.salk.edu/RiceGE/RiceGE_Data_Source.html), CSIRO (http://www.csiro.au/pi), EU-OSTID (http://orygenesdb.cirad.fr/) and ZJU-T-DNA (http://signal.salk.edu/RiceGE/RiceGE_Data_Source.html). In total, 216,942 FSTs have been identified and mapped to the rice genome. In addition, we added the information on the identification and analysis of 29,624 FSTs with Ds insertions in Additional file 1: Table S1. Thus, 246,566 FSTs were analyzed in this study (Table 1). These FSTs indicate mutations in 29,624 (53.1 %) of 55,801 genes identified by the rice genome annotation project (RGAP) annotation version 7 (Fig. 1). We defined rice genes with FSTs as “rice FST genes.” The list of FST genes with related gene-indexed mutants is provided in Additional file 1: Table S1. A total of 16,937 loci had a transposable element (TE) based on RGAP annotation version 7, and we found that 4,353 of these TE genes were tagged by mutagen insertions, such as T-DNA, Ds, dSpm and Tos17. Therefore, the mutation ratio was 25.7 % (Fig. 1) for TE genes. In contrast, 25,275 of the 38,869 non-TE genes (65 %) were tagged by mutagen insertions (Fig. 1). Previous studies indicated that mutagens such as T-DNA and transposons preferentially integrates into the transcriptionally active regions (Kim and Veena 2007). Thus, the lower proportion of insertion mutants for the TE genes suggests that a significant portion of TEs might not be actively expressed.

**Table 1:** Summary of FST genes in rice according to the individual institute in the International Rice Functional Genomics Consortium (IRFGC)

Institute	No. of FSTs	No. of mapped FSTs	No. of FSTs with insertion in genic region (55,986^a^)	No. of genes with FSTs in genic region
DJ-Ds-seq	32,459	32,009	22,002	6,841
Indica-Ds-seq	2,499	2,464	1,539	641
Affjp-Tos17	18,024	17,939	14,554	3,602
Cirad-T-DNA	29,262	27,870	17,709	5,629
csiro-Ds	611	585	398	286
Gsnu-Ds	1,072	1,050	732	484
Ostid-Ds	1,315	1,290	814	672
PFG_FSTs-T-DNA	107,171	105,739	59,707	20,889
rifgp-T-DNA	741	689	359	156
rmd-T-DNA	33,197	31,892	16,219	6,641
ship-T-DNA	12,614	9,385	4,808	1,340
trim-T-DNA	11,799	11,135	6,029	3,711
Ucd-Ds/Dspm	17,730	16,825	7,556	3,088
Total	268,496	258,872	152,426	29,664

^a^indicates the total number of loci annotated by the rice genome annotation project team (version 7.0)

### Integration of T-DNA or transposons in transcriptional active regions is evident from expression level of Agrobacterium infected calli samples

Agrobacterium mediated transformation is traditional method for transient and stable genetic transformation because of several advantages (Hiei et al. 1994). Time series microarray experiment on Agrobacterium infected rice calli revealed high transformation efficiency of japonica cultivars compared to that of indica (Tie et al. 2012). Transcriptome data generated by Tie et al. (2012) under the infection of rice calli using a super-virulent Agrobacterium tumefaciens strain *EHA 105* that containing a binary vector pCAMBIA1301 with hygromycin-resistance gene (*hpt*) and the intron-*gus* in the T-DNA region provided a good source for analysis of T-DNA insertion behavior in the rice genome. Hence, we adopted the Tie et al. (2012) dataset for our investigation. We first analyzed the distribution of non-TE genes according to their expression levels in calli, corresponding to the co-cultivation stage of Agrobacterium tumefaciens-mediated transformation process from rice Affymetrix microarray data (Tie et al. 2012). For the co-cultivation, pre cultivated embryogenic calli of a specified size were infected with suspension of Agrobacterium with an optical density (OD) of 0.35–0.4 at 600 nm for 30 min (Tie et al. 2012). The distribution data of non-TE genes based on calli expression levels are summarized in Fig. 1. The Affymetrix array has probes for 32,101 of 38,869 non-TE genes, which excludes chloroplast, mitochondria and unmapped genes from the total non-TE genes, and 22,207 of 25,275 non-TE genes with FSTs were analyzed (Fig. 1). Of the latter, there are 1,288 genes with less than 2.5 average log_2_ intensity of which 52.25 % (673 genes) have gene-indexed mutants, 9,621 genes with average log_2_ intensity from 2.5 to 5 of which 58 % (5,574 genes) have gene-indexed mutants, 7,970 genes with average log_2_ intensity from 5 to 7.5 of which 68.7 % (5,475 genes) have gene-indexed mutants, 6,140 with average log_2_ intensity from 7.5 to 10 of which 78.84 % (4,841 genes) have gene-indexed mutants, and 7,082 with more than 10 average log_2_ intensity of which 79.7 % (5,644 genes) have gene-indexed mutants (Fig. 1). In addition, 3,068 (45.3 %) of the 6,768 non-TE genes not on the rice Affymetrix array had gene-indexed mutants (Fig. 1). This ratio is even lower than that of the least expressed non-TE genes (i.e., less than 2.5 average log_2_ intensity), indicating that non-TE rice genes not printed on the rice Affymetrix gene chip are generally expressed at very low levels. On the other hand, almost 80 % of highly expressed non-TE genes had insertion mutations. Our results confirm that the more highly expressed genes in rice calli at co-cultivation stage are more likely to have mutations inserted by T-DNA, *Ds*, *dSpm* or *Tos17* (Kim and Veena 2007).

Transformation process involving T-DNA integration to the genome is a complex process and differs significantly depending on factors such as genotypes, tissue being inoculated, vector and bacterial strains, marker genes and other related culture conditions (Tie et al. 2012). Plant genes facilitate the specific transformation steps including bacterial attachment, T-DNA transfer and cytoplasmic trafficking and integration via nuclear targeting (Citovsky et al. 2007). Differentially expressed genes (DEGs) catalog of Agrobacterium mediated transformation calli to their corresponding non-treated samples is helpful to identify candidate genes responsible for T-DNA or transposons integration into genome based on expression level. So, we analyzed DEGs of Agrobacterium infected embryogenic calli of *japonica* cultivar Nipponbare and *indica* cultivar Zhenshan 97 at various time points post infection compared to uninfected calli (Tie et al. 2012). Subsequently, 4391, 4633, 3774 and 3606 probes showed differential expression with log2 fold changes > 2 and *p*-values <0.05 at 1 h, 6 h, 12 h and 24 h after infection, respectively for Nipponbare callus. Excluding multiple probes for single gene and unmapped probes to rice genome, 3095, 3613, 2926 and 2774 unique DEGs were identified for Nipponbare callus. Similarly, 4220, 3906, 3013 and 2733 DEGs were identified for Zhenshan 97 cultivar. List of DEGs and related details is presented in Additional file 2: Table S2.

### Current understanding of diverse metabolic and regulatory pathways using 1,022 rice genes with known functions

Based on information from the Overview of Functionally Characterized Genes in Rice online database (OGRO, http://qtaro.abr.affrc.go.jp/ogro/table) (Yamamoto et al. 2012), we retrieved 1,022 functionally characterized RGAP loci and mapped them to MapMan overviews (Additional file 3: Figure S1). After mapping, 1,014 genes were assigned to different MapMan terms (bins), with some candidates assigned to more than one term, resulting in 1,069 mappings (Additional file 4: Table S3). Of them, 863 genes have FSTs.

In terms of overviews, 163 genes were mapped to the metabolism overview, 455 genes to the regulation overview, 125 genes to the cellular response overview, and 44 genes were classified under large enzyme families (Additional file 3: Figure S1). The remaining mapped genes were assigned to other type of functional categories (Additional file 4: Table S3). The main subclasses of metabolism-related genes are associated with photosynthetic components and carbohydrate metabolism, cell wall metabolism, lipid metabolism, N-metabolism, amino acid metabolism, nucleotide metabolism and secondary metabolism. Among 455 genes in the regulation overview, 222 genes were assigned a role in transcription regulation. Others were related to post-transcriptional regulation, including 53 genes in protein post-translational modification, 41 genes in protein degradation, 59 genes in hormone metabolism and 64 genes assigned to various signaling events. The distribution of the characterized genes in different MapMan functional categories is provided in Additional file 3: Figure S1. In total, the functions of around 2.6 % (1,022) of non-TE rice genes (38,869) have been characterized. Functional redundancy by gene duplication and compensating metabolic pathways as well as soma-clonal variations that occurred during Agrobacterium-mediated transformation might explain why such a small portion of rice genes were functionally characterized with gene-indexed mutants. In addition, not enough biological information is available to further functionally characterize individual genes.

### Identification of FST genes with unknown function using MapMan analysis

**Fig 2:**
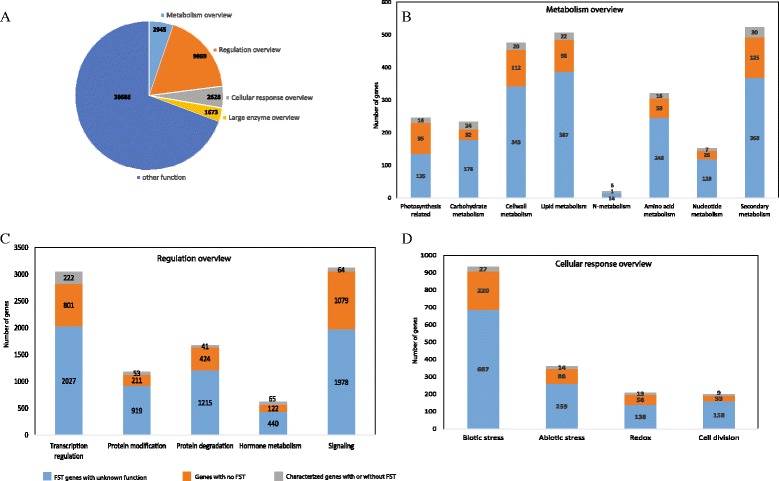
MapMan classification of all RGAP annotated genes, FST genes, and non-FST genes. **a** Proportion of FST genes mapped to four main MapMan overviews and others. Genes allotted to various sub-functional classes of main overviews for **b** metabolism overview, c Regulation overview, and **d** Cellular response overview. Each distribution bar indicates the proportion of characterized genes, FST and non-FST genes with color codes

We classified all RGAP-annotated loci using MapMan terms and determined the relative proportion of FST genes, non-FST genes and characterized genes (Fig. 2). Out of 55,801 RGAP-annotated genes, 9,869 genes fell under the regulation overview, 2,945 genes were assigned to the metabolic overview, 2,628 genes were under the cellular response overview, 1,673 genes were classified as large enzyme families and the remaining 38,686 fell either in a different MapMan overview or did not have assigned MapMan terms (Fig. 2a). In the metabolism overview, 523 genes were assigned to a secondary metabolism-related function, including 30 characterized genes, 125 non-FST genes and 368 FST genes with no functional data. Including genes with multiple functional assignment, a higher proportion of genes were assigned to lipid metabolism (507 genes) and cell wall metabolism (475 genes) (Fig. 2b). Only 21 genes were assigned to N-metabolism-related functions, and the remaining assignments were amino acid metabolism (321 genes), nucleotide metabolism (152 genes), carbohydrate metabolism (234 genes) and photosynthesis-related events (246 genes). Among genes under the metabolism overview, 76.9 % can be analyzed using FST lines. From the regulation overview, we identified 3,121 signaling, 3,050 transcription, 1,680 protein degradation, 1,172 protein modification and 627 hormone metabolism genes (Fig. 2c). Transcription (222/3050) and hormone metabolism (65/627) are more studied than others, indicating that these are major targets for regulation processes. However, 95.3 % of regulation overview genes should receive more attention through further studies, and FST lines can be utilized for 71.4 %. The majority of cellular response overview genes were attributed to biotic stress (934 genes), abiotic stress function (361), redox (209) and cell division (200) (Fig. 2d). Among these, biotic and abiotic stress are more studied than others. However, functions for 95.3 % of genes in this category should be characterized through further studies, and FST lines might be useful for 75.2 % of them.

### Functional classification of FST genes using MapMan analysis

The current understanding of the diverse metabolic and regulatory pathways with functionally characterized genes remains quite limited as shown in Additional file 3: Figure S1. Rice genes with FSTs are very useful resources for global functional genetic studies. Missing gaps in metabolism, transcription, protein modification, protein degradation, signaling and hormone pathways might be unlocked by functional genomic studies using FST mutants covering 65 % of non-TE genes. To systematically assign functions to 29,624 rice FST genes, we used the diverse overview tools installed in MapMan.

MapMan terms were assigned for 16,169 genes; no functional assignment was done for the other 13,455 genes due to a lack of matching MapMan terms. The information for all genes mapped to different overviews and the relationship to FST lines is presented in Additional file 5: Table S4. This summarized information offers a useful source of categorized candidate genes for systematic functional analyses of common metabolic/biochemical pathways or regulatory pathways, whereas genomic annotation in RAP-DB or MSU RGAP projects do not provide systematic information associated with putative function assigned to a gene. If we can use all mutants with FST genes assigned to a pathway, we might quickly expand our understanding on the pathways.

**Fig 3:**
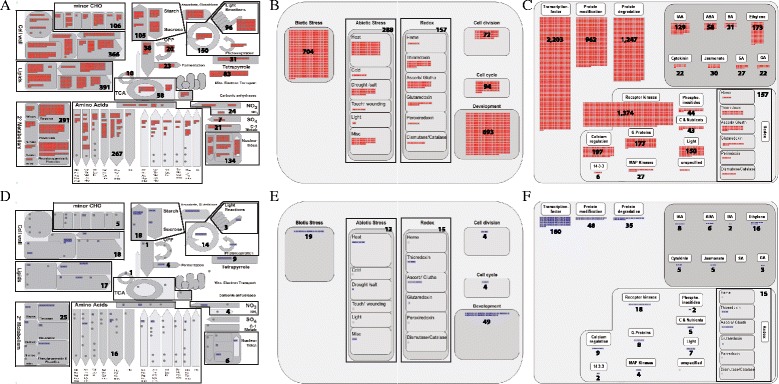
MapMan analysis of FST genes and characterized genes in metabolism, cellular response and regulation overviews. Metabolism (**a**, **d**), cellular response (**b**, **e**), and regulation (**c**, **f**) overview diagrams associated with FST genes (red boxes) and characterized genes (blue boxes). Numeric numbers indicates the number of FST genes or characterized genes mapped to sub-functional classes in each overview

Among the classified genes, 2,228 FST genes were mapped to the metabolism overview, which consists of 2,927 genes; 7,104 to the regulation overview, which consists of 9,959 genes; 1,990 to the cellular response overview, which consists of 2,643 genes; and 1,242 to the large enzyme families overview, which consists of 1,679 genes (Fig. 3; Additional file 5: Table S4). More than 70 % of these overviews can be analyzed by using FST genes.

Metabolism related genes are further divided as: 366 genes for cell wall metabolism, 391 for lipid metabolism, 391 for secondary metabolism, 267 for amino acid metabolism, 96 for light metabolism, 105 for major carbohydrate metabolism, 106 for minor carbohydrate metabolism, 150 for photosystems, 38 for glycolysis, 23 for fermentation, 10 for gluconeogenesis, 20 for oxidative phosphorylation, 58 for the tricarboxylic acid (TCA) cycle, 7 for S-assimilation, 134 for nucleotide metabolism, 21 for C1-metabolism, 16 for polyamine metabolism, 55 for co-factor and vitamin metabolism, 31 for tetrapyrrole synthesis, 83 for mitochondrial electron transport, 24 for N-metabolism, 48 for metal handling, and 53 for biodegradation of xenobiotics (Fig. 3; Additional file 5: Table S4).

Cellular response overview classified 704 genes for biotic stress, 288 for abiotic stress, 157 for redox, 693 for development, 72 for cell division, and 94 for the cell cycle (Fig. 3; Additional file 5: Table S4).

In the regulation overview, we identified 2,203 TFs, 962 genes for protein modification, 1,247 for protein degradation, 493 for hormone metabolism, and 2,038 signaling-related genes (Fig. 3; Additional file 5: Table S4). Hormone metabolism was further classified as 129 genes associated with IAA, 58 with abscisic acid (ABA), 173 with ethylene, 31 with brassinosteroid (BR), 22 with cytokinin, 30 with jasmonate (JA), 27 with salicylic acid (SA), and 22 with gibberelin (GA) (Fig. 3; Additional file 5: Table S4). The signaling-related genes were further classified as 1,374 genes associated with receptor kinases, 197 with calcium regulation, 177 with G-regulation, 6 with 14-3-3 protein, 27 with MAP kinases, 150 with light regulation, 43 with sugar and nutrient physiology, 44 with phosphoinositides, and 157 with redox-related genes (Fig. 3; Additional file 5: Table S4).

**Fig 4:**
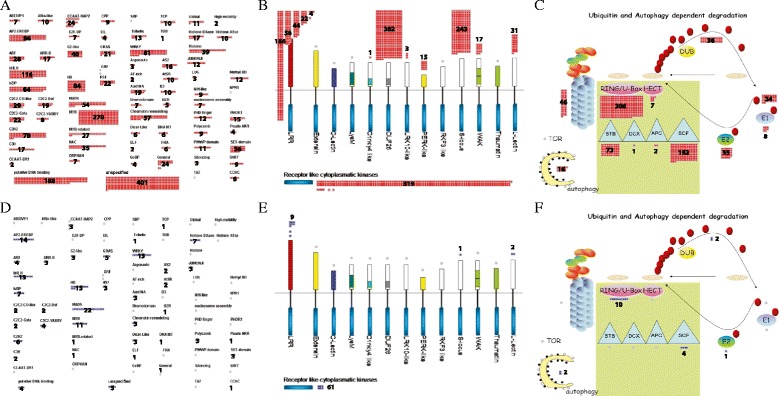
MapMan analysis of FST genes and characterized genes in transcription, kinases, and ubiquitin and autophagy dependent protein degradation overviews. transcription (**a**, **d**), kinases (**b**, **e**), and ubiquitin and autophagy dependent protein degradation (**c**, **f**) overview diagrams associated with FST genes (red boxes) and characterized genes (blue boxes). Numeric numbers indicates the number of FST genes or characterized genes mapped to sub-functional classes in each overview

Regulation pathways are coupled with transcriptional regulation; protein modification, including phosphorylation; and protein degradation. We provided detailed information on FST genes for transcriptional regulation using the transcription overview. Transcription overview consists of transcription factors and epigenetic regulation factors. For the transcription factors, family distribution of FST genes are: 270 MYB TFs, 116 basic helix-loop-helix (bHLH) TFs, 86 APETALA2/ethylene-responsive element binding protein (AP2/EREBP) TFs, 78 C2H2 TFs, 29 C2C2-CONSTANS-like TFs, 22 C2C2-GATA TFs, 19 C2C2-Dof TFs, 17 C3H TFs, 8 C2C2-YABBY TFs, 81 WRKY TFs, 84 homeobox TFs, 84 bZIP TFs, 54 MADS TFs, 35 NAC TFs, 40 G2-like/GARP TFs, 17 Arabidopsis response regulator (ARR) TFs, 4 pseudo-ARR TFs, 21 GRAS TFs, 28 auxin response factor (ARF) TFs, 19 Aux/IAA TFs, 24 CCAAT TFs, 22 heat-shock transcription factors (HSFs), 16 asymmetric leaves 2 (AS2), 10 TCP TFs, 10 B3 TFs, 10 alfin-like TFs, 10 zf-HD TFs, 10 AtSR TFs, 9 CPP1-related TFs, 7 ABI3/VP1-related B3-domain TFs, 6 Zn-finger (CCHC) TFs, 7 bromodomain TFs, 7 orphan TFs, 7 E2F/DP TFs, 6 forkhead-associated domain (FHA) TFs, 4 EIN3-like TFs, 4 GeBP like TFs, 2 AT-rich interaction domain TFs, and 2 early flowering 3 (ELF3) TFs. We also identified FST genes related to epigenetic regulation factors such as argonaute, chromatin remodeling factors, DNA methyltransferases, histone acetyltransferases, histone deacetylases, JUMONJIs, methyl binding domain proteins, nucleosome/chromatin assembly factor groups, PHD finger TFs, polycomb groups, PWWP domain proteins, SET-domain TFs, silencing groups, SNF7, and triple-helix (Trihelix) TFs (Fig. 4; Additional file 5: Table S4).

For protein modification including phosphorylation, we used the receptor-like kinase (RLK) overview and identified FST genes for 382 DUF26 RLKs, 313 LRR kinases, 243 S-locus RLKs, 31 legume lectin RLK, 15 PERK-like, 3 LRK10-like, 1 Crinkly-like, and 319 receptor-like cytoplasmic kinases (Fig. 4). LRR kinases were further classified as 186 genes associated with LRR kinase XI, 56 with LRR kinase III, 44 with LRR kinase XII, 22 with LRR kinase VIII, 4 with LRR kinase X, an LRR kinase with LRR II, and an LRR kinase with LRR VI. In general, RLKs are surface localized and transmembrane proteins consisting of an amino-terminal extracellular domains and carboxyl-terminal intracellular kinase domains (Shiu and Bleecker 2001).

For protein degradation, we used the ubiquitin and autophagy dependent degradation overview and identified 34 ubiquitins, 8 E1 ligases, 35 E2 ligases, 539 E3 ligases, 36 ubiquitin proteases, 46 proteasome complex members, and 16 autophagy-related genes (Fig. 4). The E3 ligases were further divided into 306 genes with Ring/U-box; 152 with Skp, Cullin, and F-box containing (SCF) complex; 73 with bric a brac, tramtrack, and broad complex/DDB1- CUL4-X-box/anaphase promoting complex (BTB/DCX/APC); and 7 with homologous to the E6AP carboxyl terminus (HECT).

### Gene ontology assignment for FST genes

To provide putative function for some of 13,455 FST genes which have no MapMan terms and additional information of FST genes, we assigned gene ontology (GO) terms to the rice FST genes using CARMO resource (http://bioinfo.sibs.ac.cn/carmo/) (Wang et al. 2015) in terms of biological process, molecular function and cellular components. GO analysis revealed 685 Biological process (BP) terms for 21,123 FST genes excluding multiple GO term assignment. Among the BP, metabolic process (GO:0008152) is highly enriched term and other prominent GO terms were cellular process (GO:0009987), biosynthetic process (GO:0009058) and response to stress (GO:0006950). Similarly, GO assignment revealed 368 Molecular function (MF) terms for 20,383 FST genes including binding activity related enriched terms like protein binding (GO:0005515), nucleotide binding (GO:0000166) and catalytic activity (GO:0003824). 19,399 unique FST genes were assigned to 135 GO terms in the Cellular component (CC) and the term membrane (GO:0016020) is the most overrepresented in CC. Other enriched terms include plastid (GO:0009536), nucleus (GO:0005634), plasma membrane (GO:0005886), cytoplasmic membrane-bounded vesicle (GO:0016023) and mitochondrion (GO:0005739). All FST GO term detailed information is summarized in Additional file 6: Table S5.

### Application of a rice FST gene to functional genomics study

In many cases, expression patterns of FST genes are useful indicators for further functional studies based on the analysis for known genes. Mutants with confirmed phenotypes offer potentially valuable resources to expand the current knowledge and suggest new hypotheses related to molecular mechanisms. Previously, we identified a number of mutants showing defects in light responses (Jung et al. 2008). Among them, *DXR* plays roles in the methyl-D-erythritol 4-phosphate pathway (MEP) and its mutation causes chlorine phenotypes, indicating its significance for light response in rice. In Arabidopsis, *dxr* mutants are albinos with dwarf phenotype, and those mutants were abnormal in stomata closure in leaves, have less trichrome and are unbolted. It was also revealed in the study that disruption in the MEP pathway leads to deficiency in the biosynthesis of photosynthetic pigments and phytohormones GAs and ABA (Xing et al. 2010). Here, we analyzed and proposed possible regulatory pathway for *dxr* dependent light response by integrating the transcriptome analysis and MapMan overview. Even in the absence of an in depth functional validation of the mutant, the transcriptome based MapMan analysis helps us to rapidly identify the potential candidate genes and pathways associated with *DXR*.

**Fig 5:**
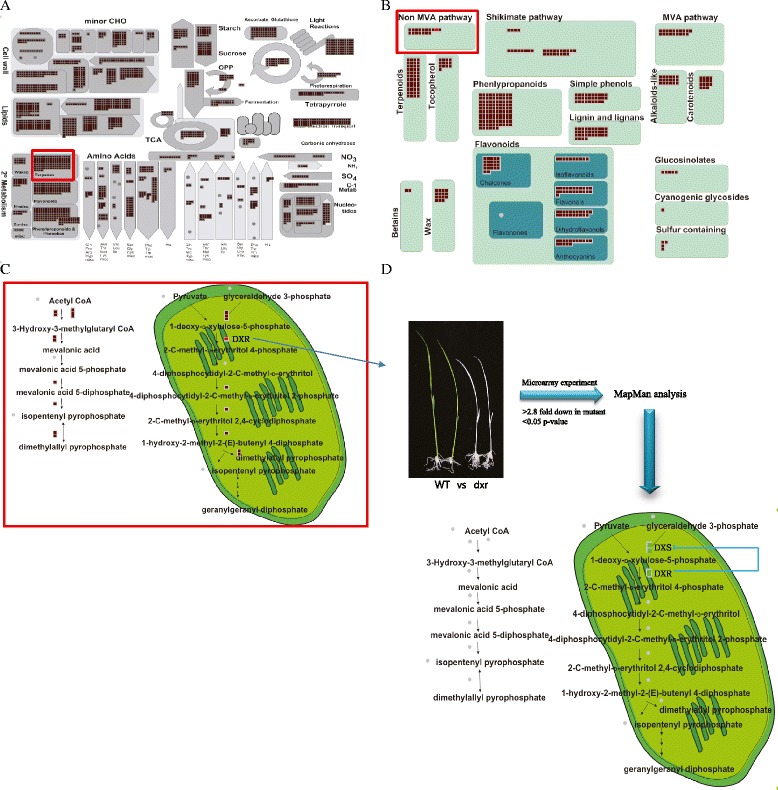
Strategy for the application of FST genes: A case study using FST mutant for *DXR* gene and transcriptome data. **a** Metabolism overview associated with FST genes (brown boxes) including *DXR* gene (red box). **b** Secondary metabolism overview associated with FST genes including *DXR* gene. **c** MEP pathway overview associated with FST genes including *DXR* gene. **d** Experimental procedure to identify downstream metabolic pathway and regulatory elements of *DXR* through microarray comparison of *dxr* mutant vs wild type. As a result, 756 downregulated genes in the *dxr* mutant compared to wild type were mapped to MEP pathway overview. Two *DXS* genes and *DXR* gene were downregulated (green boxes) in the *dxr* mutant

To do this, we first used MapMan metabolic overview analysis and identified that *DXR* gene belongs to terpene class of secondary metabolism. More detailed analysis for secondary metabolism indicates that *DXR* functions in the second step of MEP pathway to produce the iso-pentenyl pyrophosphate (IPP) (Fig. 5a-c). Towards the identification of impaired downstream pathway and elements, we carried out microarray experiments to compare this mutant and wild type controls using the Agilent 8X60K microarray. As a result, we found 756 genes showing at least 2.8 fold down-regulation in *dxr* mutants (Additional file 7: Table S6). MEP pathway overview analysis using these downregulated candidate genes in the MapMan tool revealed that two *DXS* genes in the first step of MEP pathway were down-regulated in the *dxr* mutant, indicating that first step pathway by the block of 2^nd^ step pathway was feedback inhibited and both steps are critical for the production of the final product in the pathway (Fig. 5d). More interestingly, genes from photosynthesis pathways associated with light responses, such as the photosystems I and II, calvin cycle, and photorespiration pathways, were downregulated in *dxr* mutant. Secondary metabolisms were also significantly affected by the *dxr* mutant. Regulation overview suggests downstream regulator elements for *DXR*-mediating light response in the class of transcription factors, protein modification and protein degradation (Additional file 7: Table S6; Additional file 8: Figure S2). In addition, ethylene, auxin and jasmonic acid hormones were more related to *DXR*-mediating light response, suggesting candidate genes involved in the *DXR*-mediating cross-talk between light response and hormone regulation. These genes are good targets for further analysis, representing novel regulatory candidate genes for light response. Interestingly, analysis of MEP pathway using *dxr* mutant in Arabidopsis revealed that exogenous application of GA and ABA rescued the *dxr* mutant phenotypes (Xing et al. 2010). The use of FST gene (*DXR*), relating gene indexed mutant (*dxr*), and genome-wide transcriptome analysis combined with MapMan tool will be an effective approach to quickly expand current understanding on the molecular and genetic function of FST gene which are well categorized.

## Conclusion and prospects

Distribution analysis of the affymetrix probes for FST genes based on Agrobacterium infected calli samples indicated that there is a tendency of transfer of T-DNA or transposons depend on the expression level of the gene. Although the current FSTs collection cover more than 70 % of mildly or highly expressed genes, functional characterization is still limited due to functional redundancy or a lack of guiding information for gene function. The functions of 863 of 1,022 known genes can be evaluated by current FST lines which are useful resources for functional genomic studies. We assigned 16,169 out of 29,624 FST genes to 34 MapMan classes which provide visual diagrams of diverse metabolic and regulatory pathway. In addition, missing information not covered by MapMan terms is provided by assigning GO terms in the biological process and molecular function categories. Development of new technology and quick accumulation of high-throughput data such as whole genome resequencing, microarray, and RNA-seq data might provide more options for functional studies of genes of interest or for improving agronomic traits. Integrating analysis tools of metabolic pathways and transcriptome data can help select key elements in a pathway. We applied transcriptome analysis result in the MapMan context to reveal the possible regulatory elements and pathways associated with *dxr* mutant. Microarray analysis of *dxr* mutant followed by MapMan assignment of pathways suggested a feedback inhibition mode of regulation in the MEP pathway, which still requires conformation by functional studies. In addition, mutation of *DXR* repressed photosynthesis and pathways of light reaction and diverse secondary metabolites. The use of gene-indexed mutants might enable us to carry out a systematic analysis of selected downstream pathways, accelerating the understanding of mechanisms and providing us with better options for application of the results. The Kyoto Encyclopedia of Genes and Genomes (KEGG) and RiceCyc databases (http://pathway.gramene.org/gramene/ricecyc.shtml) also provide a catalog of known or predicted rice biochemical pathways (Dharmawardhana et al. 2013; Sakurai et al. 2011).

## Methods

### Distribution analysis of FST genes using microarray data of callus samples during tissue culture and identification of DEGs

We downloaded Affymetrix microarray series GSE32426 consisting of rice embryogenic calli before and after Agrobacterium infection from NCBI GEO (http://www.ncbi.nlm.nih.gov/geo/) (Tie et al. 2012). The dataset includes expression profiles from callus right before Agrobacterium infection; 1, 6, 12 and 24 h of callus infection. The raw signal intensity (CEL file) values were first normalized with MAS5.0 method using *affy* package in R language (Gautier et al. 2004) and probes were mapped to Locus ids of RGAP. For genes with multiple probes, those with a high average expression level were considered. Genes with at least 2 fold change and *p*-value <0.05 was considered as differentially expressed. K-means and KMC clustering from MeV software (http://www.tm4.org/mev.html) was used to analyze the microarray data.

### Collection of flanking sequence tags and mapping flanking sequence tags to the rice genome

We downloaded all flanking sequence tag (FST) data from POSTECH-RISD (Jeon et al. 2000), UCD (Kumar et al. 2005), GSNU (Kim et al. 2004), CIRAD (http://oryzatagline.cirad.fr/), TRIM (http://trim.sinica.edu.tw/), NIAS (https://tos.nias.affrc.go.jp/), RMD (http://rmd.ncpgr.cn/), SHIP T-DNA (http://ship.plantsignal.cn/home.do), CSIRO (http://www.csiro.au/pi), EU-OSTID (http://orygenesdb.cirad.fr/) and ZJU-T-DNA (http://www.genomics.zju.edu.cn/ricetdna.html), and then used the BLAST program to map FSTs to the rice genome annotation version 7.0 from the RGSP website (http://rice.plantbiology.msu.edu/). FSTs with more than a 100-bp match with aligned genes were assigned to a corresponding RGAP locus ID when the FST had more than 95 % identity with the genomic DNA. For FSTs with less than a 100-bp match, we assigned the RGAP locus ID when the identity was more than 99 %. The results are summarized in Additional file1: Table S1.

### MapMan analysis

To identify metabolic pathways and regulation processes associated with FST genes in rice, we used metabolic_overview and regulation_overview installed in the MapMan tool (Thimm et al. 2004). MapMan provides the mapping files for the rice (Degenkolbe et al. 2009; Howell et al. 2009), which can be retrieved locally on installation of the software. The use of MapMan for high throughput data in rice was described in our recent study (Jung and An 2012). Related mapping files for rice genes are available from MapMan software website (http://mapman.gabipd.org/web/guest/mapmanstore). For classifying FST genes, the RGAP gene model of 29,624 rice FST genes was uploaded to the MapMan tool and 16,169 rice FST genes were mapped to MapMan terms. The remaining genes were not assigned to MapMan terms due to unknown function. To indicate downregulated gene pathways in the *dxr* mutant, genes were assigned a negative number and shown in green color. To check the feature of FST genes with a known function, we also used MapMan overviews as we analyzed all FST genes (Additional file 5: Table S4). FST and non-FST genes were distinguished using different color codes. In total, 1,022 functionally characterized RGAP loci have been summarized in the OGRO website (http://qtaro.abr.affrc.go.jp/ogro) (Yamamoto et al., 2012) and were mapped with MapMan overviews (Additional file 3: Figure S1; Additional file 4: Tables S3). MapMan metabolism includes photosynthesis pathways, carbohydrate metabolism, N-dependent pathways such as amino acid metabolism, cell wall metabolism, lipid metabolism and secondary metabolism. Various overviews installed in MapMan tool kits are described in Additional file 3: Figure S1, Additional file 4: Table S3 and Additional file 5: Table S4.

### Microarray experiments and data analysis

Rice (*Oryza sativa*) seeds with T-DNA insertion in *DXR* gene, 1B-14224, and wild type segregants from this FST line germinated on Murashige Skoog (MS) medium under controlled conditions of 28 °C day/25 °C night temperatures, 8-h light/16-h dark cycle, and 78 % relative humidity after sterilization with 50 % (w/v) commercial bleach for 30 min with gentle shaking. Leaves of mutants and wild types were collected at 7 days after germination on MS media. We compared the leaves from the *dxr* mutant which causes a defect in photosynthesis and exhibits an albino phenotype, with leaves of wild type controls. Agilent 8x60K microarray platforms were used for these transcriptome analyses (Xuan et al. 2013).

## Additional files

Additional file 1: Table S1. Summary of rice FST genes and number of related gene-indexed mutants collected from IRFGC and our Ds insertional mutants. (XLSX 1494 kb) Additional file 2: Table S2. List of differentially expressed genes (DEGs) of Agrobacterium infected rice embryogenic calli at 1 h, 6 h, 12 h and 24 h for *japonica* rice cultivar Nipponbare and *indica* rice cultivar Zhenshan 97. (XLSX 1579 kb) Additional file 3: Figure S1. Functional classification of characterized genes using MapMan analysis. A) Metabolism overview, B) Cellular response overview, C) Regulation overview, and D) Large enzyme families. FST and non-FST genes are indicated using red and green colors, respectively. E) Proportion of characterized FST genes mapped to major MapMan overviews. Genes assigned to various sub-functional classes of main overviews for F) metabolism overview, G) regulation overview, and H) cellular response overview. (EPS 8995 kb) Additional file 4: Table S3. Functional classification of 1022 characterized genes using MapMan analysis. (XLSX 54 kb) Additional file 5: Table S4. Functional classification of FST genes using MapMan terms and assignment of related FST lines. (XLSX 2505 kb) Additional file 6: Table S5. Functional classification of FST genes using GO terms in biological process and molecular function categories. (XLSX 1349 kb) Additional file 7: Table S6. List of downregulated genes in the *dxr* mutant and their MapMan classification. (XLSX 45 kb) Additional file 8: Figure S2. MapMan analysis for 756 downregulated genes in the *dxr* mutant compared to wild type. A) Metabolism, B) Photosynthesis C) Secondary metabolism, and D) Regulation overview. (EPS 2683 kb)

## Abbreviations

BP: biological process
CC: cellular component
DEGs: differentially expressed genes
Ds: dissociation
DXR: 1-deoxy-D-xylulose 5-phosphate reductoisomerase
FST: flanking sequence tags
IRFGC: the international rice functional genomics consortium
KEGG: kyoto encyclopedia of genes and genomes
MEP: methyl-D-erythritol 4-phosphate pathway
MF: molecular function
NCBI: national center for biotechnology information
OGRO: overview of functionally characterized genes in rice online database
RAP-DB: rice annotation project database
RGAP: rice genome annotation project
TFs: transcription factors
TE: transposable element
